# Congenital heart disease and arrhythmia disorders in newborns with congenital diaphragmatic hernia: a 23-year experience at a UK university pediatric surgical centre

**DOI:** 10.1007/s00383-024-05927-2

**Published:** 2024-12-19

**Authors:** Wan Teng Lee, Chun Sui Kwok, Paul D. Losty

**Affiliations:** 1 Institute of Systems, Molecular and Integrative Biology, University of Liverpool, Liverpool, UK; 2 Department of Paediatric Surgery, Alder Hey Children’s Hospital, Liverpool, UK; 3 Department of Paediatric Surgery, Ramathibodi Hospital, Mahidol University, Bangkok, Thailand

**Keywords:** Congenital diaphragmatic hernia, Pulmonary hypertension, Congenital heart disease, Cardiac arrhythmias, Wolff-Parkinson-White syndrome, Newborns, Outcomes

## Abstract

**Purpose:**

Congenital diaphragmatic hernia (CDH) is associated with congenital heart disease (CHD) and index newborns reportedly may experience cardiac arrhythmia disorders [Tella et al.—Pediatric Critical Care Medicine 2022]. This study analyses, details and reports contemporary outcome metrics of CHD and cardiac rhythm disease (CRD) in CDH babies attending a university surgical centre.

**Methods:**

Retrospective analysis of medical records of all newborns undergoing Bochdalek CDH repair between 1999 and 2021 at a university paediatric surgical centre. CDH newborns with CHD and neonatal arrythmias were identified from echocardiogram and electrocardiogram (ECG) investigative studies. Operative native diaphragm and / or use of patch repair(s) was documented. Outcome(s) measured—(i) mortality and (ii) cardiopulmonary interventions including ventilatory strategies—ECMO (%), inotropes and anti-arrhythmic therapy(s).

**Results:**

Of 173 CDH neonates, 95 (55%) had CHD of which 9 babies (10%) had cardiac arrhythmias. CDH and co-existing CHD was linked with (a) lower infant birth weights (3130 g vs 3357 g, *p* = 0.05), (b) increased use of inotrope agents (48.4% vs 39.3%, *p* = 0.03) and (c) greater use of high-frequency oscillatory ventilation (38.9% vs 23%, *p* = 0.004). CDH babies experiencing arrythmias were at higher risk (%) of developing pulmonary hypertension (66.7% vs 28.7%, *p* = 0.01). No significant differences were observed in ECMO utilisation (12% vs 6%, *p* = 0.46) or patch repair(s) (53% vs 46%, *p* = 0.06) in CDH patients with and without CHD. CHD was not associated with increased risk(s) of mortality (OR 2.58, 95% CI 0.81–8.24, *p* = 0.11). Of 9 index CDH patients with arrhythmias—4 babies (44%) required interventional treatments.

**Conclusion:**

CHD was prevalent in a high percentage (%) of CDH newborns treated at this university centre and associated with increased use (%) of cardiovascular respiratory support including patch repair. A minority of patients (2.3%) had cardiac rhythm disorders requiring treatment(s). In those developing arrhythmias pulmonary hypertension may be a risk-linked event. Optimising outcomes to offset pulmonary hypertension requires further appraisal. Future large-scale population studies may help underscore the ‘real apparent incidence’ of cardiac rhythm disorders in CDH.

## Introduction

Congenital diaphragmatic hernia (CDH) is notably linked with other associated malformations of which congenital heart disease (CHD) may be encountered in some 10%–28% index cases [1–3]. Cardiac lesions vary in severity ranging from what may be considered ‘non-significant’ subtypes such as patent foramen ovale (PFO) and patent ductus arteriosus (PDA) to critical major structural malformations such as Tetralogy of Fallot and hypoplastic heart syndrome amongst several other variants [1, 3]. Despite recent advances which now include prenatal diagnoses, elective delivery at specialist centres, improved perinatal care management and extracorporeal membrane oxygenation (ECMO), CDH newborns with cardiac malformations may experience a higher mortality (%) and morbidity compared to those babies without CHD [1–5].

In addition to cardiac malformations, heart arrhythmia disorders potentially can also impact outcomes of CDH patients such that their incidence (%) and reporting in index cases remains to be fully investigated [6, 7]. A recent study by Tella et al. in 2022 from Boston Children’s Hospital USA interestingly reported that 11% of CDH newborns reportedly experienced supraventricular tachycardia (SVT) events during their initial hospitalisation stay [6]. SVT occuring in these CDH newborns was associated with structural heart disease, ECMO usage, longer hospital stay and home oxygen dependency on discharge [6]. The current study considered it instructive therefore to analyse, detail, and report contemporary outcome metrics of CHD and cardiac rhythm disease (CRD) in CDH babies attending a UK university paediatric surgical centre.

## Methods

### Study cohort

A retrospective chart review of medical files and electronic patient case records of all neonates who underwent Bochdalek CDH repair between January 1999 and December 2021 at Alder Hey Children’s Hospital, Liverpool, UK, was conducted. Patients with Morgagni CDH and late CDH diagnoses were excluded.

### Identification of CHD, major CHD, and arrhythmias

CDH newborns with congenital heart disease (CHD) were identified from echocardiography investigative studies by a multidisciplinary team of paediatric cardiologists and attending neonatologists. Descriptors of CHD were detailed, analysed, and subcategorised based on cardiac defect type(s). Minor cardiac lesions including PFOs and PDA(s) were included in full audit analyses but the main emphasis of focus was particularly extended to CDH newborns who had major structural CHD. Heart arrhythmias events were identified from electrocardiogram (ECG), 24-h ECG tracings and the informatics obtained from medical chart case notes and electronic patient records (EPR) data verified by the paediatric cardiology and neonatology service teams.

### Clinical characteristics and outcomes

Data collected included gestational age, sex, birth weight, syndromic disease, laterality / side of CDH defect and whether CDH was diagnosed antenatally. Clinical care recorded the use of inhaled nitric oxide (iNO), high-frequency oscillatory ventilation (HFOV), ECMO, primary native versus patch CDH repair(s) from surgeon operative records and anti-arrhythmic cardiac intervention(s) where required. Outcome parameters studied and also detailed were (i) presence of pulmonary hypertension (%), (ii) oxygen dependency on hospital discharge and (iii) mortality (%).

### Statistical analyses

Baseline clinical characteristics, interventions and patient outcomes were compared between (a) CDH patients with and without CHD; (b) CDH newborns with and without major CHD malformations, and (c) CDH index cases with and without those who had cardiac arrhythmias. Normality of distributions was assessed using Kolmogorov–Smirnov and Shapiro–Wilk tests. Fisher’s exact test was used for comparison of all categorical variables whilst the Mann–Whitney U-test was used for continuous variables. Categorical variables were expressed as count (%) whilst continuous variables were expressed as median (interquartile range). All statistical analyses were conducted using IBM SPSS Statistics software version 29.0.2.0 (Armonk, NY) with statistical significance defined as *p* < 0.05.

## Results

### Baseline data characteristics

Between January 1999 and December 2021, 173 index CDH neonates were treated at Alder Hey Children’s Hospital Liverpool UK. Ninety five (55.0%) CDH newborns had CHD of which 39 cases (22.5%) were defined as having major structural cardiac malformations. Nine (5.2%) CDH babies experienced at least one or more episode(s) of heart arrhythmia disorder(s). Left-sided Bochdalek CDH defects were the most common diaphragm phenotype anomaly documented in 137 (79.1%) newborns—(Table 1).

**Table 1:** Baseline data characteristics of all CDH neonates. Categorical variables are expressed as count (%) and continuous variables expressed as median (interquartile range)

Baseline data characteristics	Total (*n* = 173)
Gestational age (weeks)	38 (37–40)
Birth weight (g)	3240 (2801–3615)
Male	109 (63.0%)
Antenatal diagnosis	74 (42.8%)
Syndromic disease	23 (13.3%)
Left sided CDH Bochdalek defect	137 (79.1%)
All congenital heart disease	95 (55.0%)
Major congenital heart malformations (PFO, PDA excluded)	39 (22.5%)
Arrhythmias	9 (5.2%)

### Variant types of congenital heart disease

The spectrum of cardiac disease in the CDH patient study cohort is listed in Table 2. Shunt-related CHD was the most common CHD documented in 85 (89.4%) CDH newborns. After excluding PFO and PDA—isolated atrial septal defects (ASD) featured as the most prevalent major CHD lesion in 15 (15.8%) CDH newborns. The remaining patients all had similar frequency distributions (%) of major CHD ranging from duct-dependent defects notably aortic coarctation (2.1%) and Tetralogy of Fallot (1.0%) to valvular CHD namely aortic valve (1.0%) and tricuspid regurgitative lesions (1.0%).

**Table 2:** Congenital heart disease and arrhythmia(s) by type expressed as count (%)

Congenital heart disease	Total (*n* = 95)
Duct dependent—systemic circulation	4 (4.2%)
Aortic coarctation	2 (2.1%)
Hypoplastic aortic arch syndrome	2 (2.1%)
Duct dependent—pulmonary circulation	3 (3.2%)
Pulmonary atresia or stenosis	2 (2.1%)
Tetralogy of Fallot	1 (1.0%)
Shunts	85 (89.4%)
Ventricular septal defect (VSD)	2 (2.1%)
Atrial septal defect (ASD)	15 (15.8%)
Atrioventricular septal defect (AVSD)	1 (1.0%)
PFO	60 (63.2%)
PDA	52 (54.7%)
Valvular	3 (3.2%)
Hypoplasia of pulmonary valve	1 (1.0%)
Bicuspid aortic valve	1 (1.0%)
Aortic regurgitation	1 (1.0%)
Tricuspid regurgitation	1 (1.0%)
Other structural lesion heart disease	4 (4.2%)
Rhabdomyoma	1 (1.0%)
Dextrocardia	2 (2.1%)
Large pericardial effusion	1 (1.0%)
Arrhythmias	9 (9.5%)
Supraventricular tachycardia (SVT)	5 (5.3%)
Recurrent sinus tachycardia(s)	3 (3.2%)
Wolff-Parkinson-White syndrome (WPW)	1 (1.0%)

Of 9 CDH babies who experienced heart arrhythmias, these comprised 5 (5.3%) patients with SVT, 3 (3.2%) patients with recurrent episodes of sinus tachycardia(s) and a single patient (1.0%) with the Wolff-Parkinson-White syndrome conduction system disorder.

### Comparison of CDH patients with and without congenital heart disease (CHD)

Differences in the clinical characteristics examining CDH patients with and without CHD are listed in Table 3. Compared to CDH newborns without CHD, those with CHD had notably lower birth weight(s)—(3130 g vs 3357 g, *p* = 0.053). CDH babies with CHD required greater intensive inotropic support (48.4% vs 39.3%, *p* = 0.034) and HFOV dependency (38.9% vs 22.5%, *p* = 0.004) compared to those without CHD. Further analysing CDH patients with and without major CHD structural malformations (Table 4), those in particular with major CHD were noted to have significantly much lower birth weights (2930 g vs 3357 g, *p* = 0.011) and had notable association(s) with syndromic disease (17.9% vs 9.7%, *p* = 0.041). CDH patients with major CHD malformations had increased requirement(s) for (a) HFOV (33.3% vs 22.5%, *p* = 0.052), (b) CDH patch repairs (59.0% vs 45.2%, *p* = 0.015) and (c) home oxygen dependency on hospital discharge (12.8% vs 1.6%, *p* = 0.02). CDH newborns with major CHD had also higher rates (%) of ECMO utilisation (15.4% vs 9.7%, *p* = 0.332) with a risk trend toward a seemingly worse mortality (15.4% vs 6.5%, *p* = 0.176) although these latter data findings would prove statistically non-significant.

**Table 3:** Comparison of CDH patients with and without CDH (any)

Clinical characteristics	No CHD (*n* = 62)	All CHD (*n* = 95)	*P* value
Gestational age (weeks)	38 (37–39)	38 (37–39)	0.912
Birth weight (g)	3357 (2977–3700)	3130 (2600–3540)	0.053
Male	41 (67.2%)	56 (58.9%)	0.404
Antenatal diagnosis	27 (44.2%)	50 (52.6%)	0.128
Syndromic disease	6 (9.7%)	8 (8.4%)	1.000
Pulmonary hypertension	12 (19.4%)	40 (42.1%)	0.060
Inotropes	24 (39.3%)	46 (48.4%)	0.034
HFOV	14 (22.5%)	37 (38.9%)	0.004
iNO	13 (21.3%)	37 (38.0%)	0.067
ECMO	6 (9.7%)	12 (12.6%)	0.458
Patch repair (diaphragm, abdomen, double)	28 (45.2%)	53 (55.8%)	0.060
Mortality	4 (6.5%)	14 (14.7%)	0.079
Home oxygen dependency	1 (1.6%)	5 (5.3%)	0.235

**Table 4:** Comparison of CDH patients with and without major CDH (PFO and PDA excluded)

Clinical characteristics	No CHD (*n* = 62)	Major CHD (*n* = 39)	*P* value
Gestational age (weeks)	38 (37–39)	38.5 (38–39)	0.353
Birth weight (g)	3357 (2977–3700)	2930 (2635–3420)	0.011
Male	41 (67.2%)	20 (51.2%)	0.213
Antenatal diagnosis	27 (44.2%)	17 (43.6%)	0.833
Syndromic disease	6 (9.7%)	7 (17.9%)	0.041
Pulmonary hypertension	12 (19.4%)	13 (33.3%)	0.222
Inotropes	24 (39.3%)	17 (43.6%)	0.255
HFOV	14 (22.5%)	13 (33.3%)	0.052
iNO	13 (21.3%)	11 (28.2%)	0.286
ECMO	6 (9.7%)	6 (15.4%)	0.332
Patch repair (diaphragm, abdomen, double)	28 (45.2%)	23 (59.0%)	0.015
Mortality	4 (6.5%)	6 (15.4%)	0.176
Home oxygen dependency	1 (1.6%)	5 (12.8%)	0.020

### Comparison of CDH patients with and without heart arrhythmias

Differences in clinical characteristics between CDH patients with and without heart arrhythmias are listed in Table 5**.** Significantly more CDH patients with an arrhythmia disorder experienced pulmonary hypertension (66.7% vs 28.7%, *p* = 0.010). In contrast, though we found here no significant differences recorded in the use of inotropes, iNO, HFOV or ECMO between these two distinct groups. There were also no significant differences noted comparing mortality rate (%) between CDH patients with and without heart arrhythmia(s)—(p N.S.).

**Table 5:** Comparison of CDH patients with and without cardiac arrhythmia(s)

Clinical characteristics	No arrhythmias (*n* = 164)	Arrhythmias (*n* = 9)	*P* value
Gestational age (weeks)	38 (37–39)	38.5 (38–39)	0.536
Birth weight (g)	3220 (2880–3633)	3545 (3282 -3567)	0.332
Male	103 (62.8%)	6 (66.7%)	1.000
Antenatal diagnosis	71 (43.3%)	5 (55.6%)	0.740
Syndromic disease	12 (7.3%)	2 (22.2%)	0.188
Congenital heart disease (all)	87 (53.0%)	5 (55.6%)	1.000
Major congenital heart disease (PFO and PDA excluded)	34 (20.7%)	3 (33.3%)	0.393
Pulmonary hypertension	47 (28.7%)	6 (66.7%)	0.011
Inotropes	66 (40.2%)	5 (55.6%)	0.720
HFOV	49 (29.9%)	6 (66.7%)	0.058
iNO	47 (28.7%)	4 (44.4%)	0.175
ECMO	16 (9.8%)	2 (22.2%)	0.270
Patch repair (diaphragm, abdomen, double)	82 (50.0%)	5 (55.6%)	1.000
Mortality	18 (11.0%)	2 (22.2%)	0.325
Home oxygen dependency	9 (5.9%)	0 (0.0)	1.000

### Interventions for CDH patients with heart arrhythmias

Of 9 CDH patients with heart arrhythmias, 4 (44.4%) index cases resolved spontaneously without requiring interventions—(Table 6). Two of nine (22.2%) CDH babies experienced isolated episodes of SVT following operative defect repairs both of which were treated and resolved with a ‘one-shot’ dosage of digoxin therapy. A single patient (11.1%) had 2 isolated episodes of SVT with no underlying major cardiac structural defects detected on echocardiography and was also treated successfully with digoxin. A further CDH baby (11.1%) in the study cohort population required cardiac ablation therapy for recurrent episodes of SVT and sinus tachycardia which were linked to a final diagnosis of Wolff-Parkinson-White syndrome. Two (22.2%) deaths were recorded in this study cohort group. The first CDH baby had an aortic coarctation lesion without having undergone corrective cardiac surgery. The neonate had required both diaphragmatic and abdominal patch repair operations for CDH and had both HFOV and ECMO with intensive inotropic support during hospitalisation. The baby subsequently died from a fatal cardiac arrest due to a severe hypotensive crisis. The second patient fatality had hypoplastic aortic arch syndrome with pulmonary hypertension. The newborn had required a diaphragmatic patch repair and laparostomy silo created due to intra-operative deterioration and was placed on HFOV and inotropes post-operatively. Although the patient experienced one episode of a self-resolving SVT post-operatively, death was later due to multiple systems organ failure (MSOF).

**Table 6:** Interventions for CDH patients with cardiac arrhythmia(s)

Intervention for heart arrhythmia(s)	Total (*n* = 9)
Self-resolution or no intervention	4 (44.4%)
Medical (digoxin)	3 (33.3%)
Ablation therapy	1 (11.1%)
Deaths	2 (22.2%)

## Discussion

This current study report has examined the prevalence and outcomes of CDH patients with co-existing CHD and heart arrhythmia disorders at a UK university paediatric surgery centre. The prevalence (%) of major CHD (22.5%) in the CDH patient population at Alder Hey Children’s Hospital Liverpool is consistent with other quality reporting studies notably observed in 10%–28% CDH index cases [1–3, 8]. Atrial septal defects (ASD) were the most common CHD lesion encountered in our CDH newborn population as also shown by a previous study [9] whilst several published reports cite ventricular septal defects (VSD) to be the most common single associated cardiac malformation [1, 2, 5, 8–10]. VSD notably also features as an intrinsic component lesion of other variant CHD structural anomalies notably Tetralogy of Fallot and truncus arteriosus [10].

Left-sided CDH accounted for the most common (79.1%) phenotype defect seen in the study population and the lesion defect type is well known to disrupt fetal heart development and function in several mechanistic ways. Echocardiography studies have shown that the direct compression forces of herniated intrathoracic abdominal viscera, liver and spleen contribute to left ventricular hypoplasia, reduced-sized hilar pulmonary arteries and smaller aortic and mitral heart valves [4, 11–13]. The resultant deleterious outcome is reduced left-sided cardiac filling with subsequent left ventricular dysfunction [14]. A multicentre study has demonstrated that left ventricular dysfunction is recorded in up to 24% of CDH patients whilst another study noted that the prevalence of hypoplastic left heart syndrome was some 124 times higher in CDH versus the general population [5, 15], Together, the dual combination of left ventricular dysfunction with left heart hypoplasia is associated with the highest risk(s) of mortality and ECMO use in patients with CDH [16].

Interestingly in this current study, we found that CDH patients with major CHD had lower birth weight(s) and higher rates (%) of syndromic disease(s). This may be readily explained—in part—as many human genetic syndromes have known associations with CHD as part of their disease phenotype [17, 18]. For example, in our study cohort we had Trisomy 21 patients with AVSD cardiac malformations whilst other newborns had Pentalogy of Cantrell with both ASD and VSD lesions.

CDH patients with CHD had increased requirement(s) for intensive cardiopulmonary support and interventions including inotropes, HFOV and later home oxygen dependency. There were also trends towards increased need for ECMO and a higher mortality (%). These key findings correlate well with published studies that report a prolonged duration of mechanical ventilation, increased use of ECMO and supplemental oxygen requirements on hospital discharge in CDH patients with CHD [2, 19, 20]. Contrary to the moderately good institutional survival outcomes we cite in this current report others have shown that CDH patients with CHD record a significantly higher mortality (%) ranging between 45% and 76% cases [1, 2, 5, 21]. A further published study has noted that CDH newborns with associated major cardiac anomalies had a 102-fold increased risk of 6-month mortality than those without heart defects [22]. This is most likely related to the wide heterogeneity and complexity of significant cardiopulmonary morbidity that may be encountered in certain vulnerable CDH populations.

Most CDH newborns in our study population with major CHD underwent patch repair(s) clearly showing that these patients have more severe complex and much larger-sized phenotype defects. These study findings are corroborated and consistent with ‘big data’ from the CDH Study Group International Registry who report a higher prevalence (%) of cardiovascular malformations in the largest C and D diaphragm subtypes [19]. A higher mortality (%) without achieving defect repair of CDH was also observed in those babies with major CHD highlighting the real challenges in resuscitating and stabilising the very sickest vulnerable newborns [1, 20].

We report that 9 (5.2%) CDH newborns developed heart arrhythmias in this study population during their initial hospitalisation stay. A recent single-centre study by Tella et al. in 2022 from Boston Children’s Hospital USA has evaluated tachyarrhythmia disorders in CDH patients and found a prevalence of 11% in their cohort group [6]. The authors of this report also recorded frequent SVT events (171 episodes) amongst their CDH patients with varied underlying precipitating causes identified including serum electrolyte abnormalities and central venous catheter device malpositioning [6]. Apart from a single CDH newborn with Wolff-Parkinson-White syndrome—a cardiac system conduction disorder—most of the heart arrhythmia events in our Liverpool CDH study population occurred spontaneously with no obvious triggers found. We found only 4 of 9 (44.4%) CDH babies with heart arrhythmias in this study report required definitive treatment(s)—(Table 6) which were significantly fewer than the 71% intervention rate described by Tella et al. with their CDH patients managed at Boston Children’s Hospital USA [6].

We record in the current study that CDH patients with heart arrhythmias had notably higher rates (%) of pulmonary hypertension. Although the basic pathophysiology of pulmonary hypertension in CDH is becoming increasingly better understood, the finding(s) of heart arrhythmias and pulmonary hypertension in CDH patients is likely more complex and elusive [23–25]. A previous study on adult populations found that SVT arrhythmias were noted in 11.7% of patients with pulmonary hypertension of which some 84% of the SVT episodes were considered particularly associated with right ventricular heart failure [26]. Several mechanisms of arrhythmogenesis in pulmonary hypertension have thus been proposed. Elevated pulmonary arterial pressures observed in pulmonary hypertension result in right ventricular hypertrophy with upstream right atrial heart dilatation [27]. In consequence, fibrosis, vascular cell degeneration and infarction can later occur within the sinus and atrioventricular nodal systems leading thus onwards to heart arrhythmias [27, 28]. Other studies have postulated that pulmonary hypertension dysregulates right ventricular autonomic activity predisposing the heart to develop arrhythmias [28, 29]. Extrapolating these insightful observations from adult speciality cardiology studies it may or could indeed be interesting to undertake future investigative studies to determine whether pathogenetic events like these play a role in the fetal transitional–newborn circulation in CDH giving rise to heart arrhythmias.

In closing this study reaffirms that major CHD is prevalent (%) in a significant proportion of CDH newborns and that this is associated with an increased rate / use of intensive cardiorespiratory intervention(s) highlighting the burden of cardiorespiratory morbidity (%) experienced in a vulnerable ‘ at risk’ patient population [30, 31]. CDH patients with major CHD have notably higher requirement(s) and rates of CDH patch repair indicating a severe larger defect-sized phenotype. We further show that 10% of CDH newborns at this university surgical centre experienced cardiac arrhythmias during their initial primary hospitalisation stay and this can notably be linked to higher rates (%) of pulmonary hypertension. Optimising outcomes to offset pulmonary hypertension requires further appraisal. Whilst we acknowledge our study may have limitations from single institution reporting it will be of interest we believe to undertake future large-scale population-based studies to highlight the impact of CHD and the ‘real apparent incidence’ of cardiac rhythm disorders in CDH.

## Data Availability

No datasets were generated or analysed during the current study.
